# Artificial intelligence in pathology: a framework for preserving brain capital in the diagnostic apex

**DOI:** 10.3325/cmj.2026.67.219

**Published:** 2026-06

**Authors:** Steven N. Hart

**Affiliations:** Mayo Clinic, Rochester, MN, USA

## Abstract

**Aim:**

To present a five-criterion calibration framework for evaluating artificial intelligence (AI) tools in pathology centered on preserving “brain capital” – the finite cognitive resources available to clinicians – and stabilizing the “diagnostic apex,” the point where histological evidence meets clinical judgment.

**Methods:**

Using cognitive load theory (CLT) and implementation science, we developed the Pathology AI Diagnostic Balance – a conceptual model mapping the physician-specimen interaction within working memory and IT infrastructure constraints – and examined it in the context of peer-reviewed case studies of implemented pathology AI tools.

**Results:**

The framework identifies five criteria: Contextual Literacy (integration of clinical history), Responsibility (preservation of signing authority), Evidence Gap Analysis (closure of diagnostic uncertainty), Verification (feasibility of quality control), and Opportunity Cost (return on cognitive investment). Successful tools, such as Ki-67 quantification and breast cancer screening, reduce cognitive burden by automating routine tasks or enabling rapid verification. Conversely, tools requiring an exhaustive re-review or producing opaque recommendations deplete cognitive bandwidth, causing decision fatigue and diagnostic errors.

**Conclusion:**

Pathologists are positioned to exercise evaluative authority over tools that increase extraneous cognitive load. Clinical expertise represents pathologists’ most distinctive contribution to AI development: defining which failure modes matter clinically, what context an AI must integrate, and whether a tool serves patient care. By prioritizing cognitive sustainability over purely technical metrics, the profession can ensure AI functions as a genuine enhancement of diagnostic capability rather than an additional burden on diagnostic workflows.

Pathology stands at a critical digital crossroads, driven by converging pressures: an acute shortage of trained professionals, rising cancer incidences worldwide, and increasing complexity of diagnostic workloads (1,2). While computational pathology offers potential solutions through automation and decision support, a significant “translation gap” persists between model development and clinical implementation (3,4). Many AI tools demonstrate impressive accuracy in controlled research settings but fail to gain traction in clinical practice – not due to insufficient technical performance but due to a mismatch between tool design and clinical workflow requirements. Bridging this gap requires active physician leadership in AI evaluation. Pathologists hold knowledge that engineers cannot derive from data: which failure modes carry clinical consequence, how contextual factors shift diagnostic probability, and what “good” looks like for a patient. This represents expertise that cannot be derived from data alone and constitutes the essential ingredient that transforms technical performance into clinical value.

Current evaluations of AI tools in pathology focus predominantly on statistical measures such as area under the receiver operating characteristic curve (AUC), sensitivity, and specificity. While essential for technical validation, these metrics are insufficient for predicting clinical utility. An AI tool with 95% accuracy that requires 30 minutes of verification per case may be far less valuable than a tool with 90% accuracy verifiable in 30 seconds (5). Such metrics neglect a clinician’s “brain capital” – the finite cognitive resources available for diagnostic reasoning, decision-making, and quality assurance.

Brain capital encompasses working memory capacity, attentional focus, decision-making stamina, and the ability to integrate multiple information sources. Cognitive psychology has established that human working memory can hold only 4 ± 1 chunks of information simultaneously (6), and that prolonged effort leads to decision fatigue – a documented decline in decision quality after extended complex reasoning (7). In analogous visual-diagnostic specialties, accuracy declines measurably over a workday, with radiologist AUC falling from 0.885 to 0.852 due to cumulative cognitive fatigue (8).

Cognitive load theory (CLT) distinguishes three types of cognitive load (6): intrinsic load (inherent task complexity), germane load (productive cognitive effort building expertise), and extraneous load (unnecessary burden from poor tool design). Effective AI tools should reduce extraneous and intrinsic load for routine tasks, thereby preserving brain capital for complex diagnoses where human expertise is irreplaceable. We conceptualize the “diagnostic apex” as the critical point where histological evidence meets clinical judgment – the moment when a pathologist integrates visual findings, clinical history, and prior experience to render a diagnosis. The apex is destabilized when poorly designed AI tools add extraneous cognitive load, consuming brain capital without commensurate diagnostic value.

This article presents a five-criterion calibration framework centered on preserving brain capital at the diagnostic apex, enabling pathologists to assess whether AI tools genuinely enhance diagnostic capability or merely redistribute burden.

## Methods

### Conceptual framework development

We developed the framework through narrative synthesis of principles from CLT and implementation science. The methodological approach comprised three phases: 1) theoretical modeling of the physician-specimen diagnostic interaction using CLT as the primary framework; 2) identification of published case studies of implemented AI tools in pathology, selected from peer-reviewed literature to represent commercially deployed or prospectively validated tools with quantitative outcome data illustrating variation across the five evaluation dimensions; and 3) iterative refinement of evaluation criteria against those case studies.

**Figure 1:**
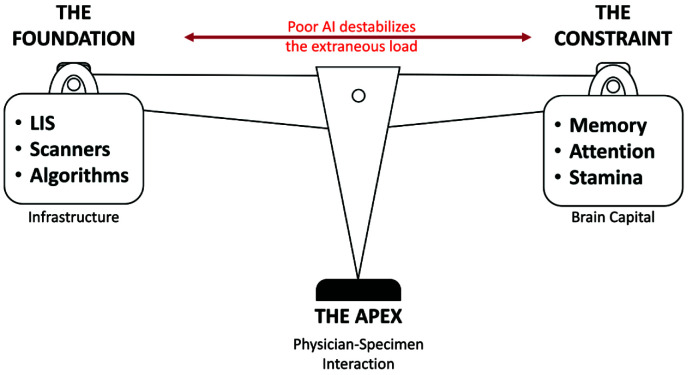
The Pathology AI Diagnostic Balance. Three interacting components of diagnostic artificial intelligence (AI) deployment are arranged as a beam balance. The foundation (left pan) represents technical infrastructure – laboratory information systems (LIS), imaging platforms, and AI deployment layers; infrastructure failures shift cognitive burden upward to the apex. The constraint (right pan) represents the universal biological limitation of working memory – finite brain capital comprising attention, stamina, and cognitive focus. The inverted fulcrum represents the apex: the physician-specimen interaction, the critical point where histological evidence meets clinical judgment. The level beam denotes equilibrium: well-designed AI tools support the foundation without imposing extraneous cognitive load at the apex. Destabilization occurs when opaque or unverifiable AI shifts excessive cognitive burden to the apex, depleting the brain capital required for diagnostic reasoning.

The AI Diagnostic Balance (Figure 1) provides the theoretical foundation by mapping three interacting components of diagnostic AI deployment as a beam balance in equilibrium. The apex, occupying the fulcrum position, represents the pathologist’s cognitive workspace – the integration point for visual findings, clinical history, laboratory data, and prior experience. Stabilization requires AI outputs that are clear, verifiable, and aligned with clinical reasoning; explainable outputs with spatial localization enable rapid verification (9), while opaque recommendations without disclosed reasoning force pathologists either to accept AI output without understanding or to conduct an exhaustive re-review, both of which deplete brain capital. The foundation, represented by one pan of the balance, represents the technical infrastructure: laboratory information systems, image management platforms, and AI deployment layers. Foundation failures shift cognitive burden upward to the apex, and system reliability is among the strongest predictors of user acceptance in digital pathology (10). The constraint, represented by the opposing pan, represents the universal biological limitation of working memory. A pathologist diagnosing complex lymphoma may need to simultaneously consider morphological patterns, immunohistochemistry results, clinical presentation, molecular findings, and relevant differential diagnoses – already at or beyond working memory capacity (6,11). Effective AI tools reduce the number of items requiring simultaneous consideration or present information in formats aligned with existing cognitive schemas.

These three components interact dynamically: a robust foundation reduces load at the apex; AI tools designed within working memory constraints keep the diagnostic balance stable. The five-criterion calibration framework operationalizes this conceptual model into practical evaluation criteria.

## Results

The five-criterion calibration framework is summarized in Table 1. Each criterion is described with its theoretical rationale, evaluation criteria, and a case study of a deployed AI tool.

**Table 1:** The five-criterion calibration framework for evaluating artificial intelligence (AI) tools in pathology. Each criterion addresses a distinct dimension of cognitive impact at the diagnostic apex

Calibration criterion	Core cognitive requirement	Case study	Key outcome metric
1. Contextual Literacy	Accept and integrate clinical context; adjust outputs accordingly	PathChat	Outperformed GPT-4V and competing vision-language models on structured pathology question-answering benchmarks (12)
2. Responsibility and Signing Authority	Spatially localized outputs enabling verification in seconds	Paige Prostate	70% reduction in cancer detection errors; 8 percentage point increase in sensitivity across all histologic grades (13)
3. Evidence Gap Analysis	Precise quantification of uncertain parameters to resolve diagnostic ambiguity	Lunit SCOPE PD-L1	32% reduction in PD-L1 reading time; concordance maintained at clinical decision thresholds (14)
4. Verification and Stress-Testing	Companion verification mechanism; published artifact stress-test data	AQuA framework	99.8% hallucination detection accuracy at 1-2 s per field (15)
5. Resource and Opportunity Cost	Net positive return on brain capital at the system level	ZAS Hospitals prostate AI	9-h TAT reduction; one-third fewer IHC studies; financial ROI within 18 months (10)

*Abbreviations: AI – artificial intelligence; IHC – immunohistochemistry; PD-L1 – programmed death-ligand 1; ROI – return on investment; TAT – turnaround time.

### Criterion 1: Contextual Literacy

Pathological diagnosis is inherently contextual: the same histological pattern may represent reactive changes in one clinical scenario and neoplasia in another. AI tools that analyze tissue images without access to clinical context create a fundamental mismatch with clinical reasoning, requiring pathologists to mentally reconcile AI output with clinical information the AI never received – extraneous cognitive load that directly depletes brain capital (16,17). Tools demonstrating contextual literacy accept and integrate structured clinical data, adjust diagnostic probabilities based on context, and flag when critical information is absent.

One published illustration of high contextual literacy is PathChat (12,18). This multimodal foundation model combines visual encoders with natural language processing, achieving superior performance compared with context-naive models. When shown atypical lymphoid proliferation with the clinical note “post-transplant patient,” PathChat appropriately weighted post-transplant lymphoproliferative disorder rather than conventional lymphoma, aligning AI output with the pathologist’s own contextual reasoning and eliminating the additional mental step of recontextualizing the AI recommendation. By contrast, pixel-only models have been documented to flag reactive germinal centers, crush artifacts, and tangential sections as suspicious for lymphoma; in one series, up to 40% of AI-generated alerts were attributable to reactive or artifactual changes that a context-aware pathologist would immediately dismiss (19). Such context blindness transforms an AI tool from a cognitive aid into a source of additional workload.

### Criterion 2: Responsibility and Signing Authority

Pathologists bear ultimate medical-legal responsibility for diagnostic reports and cannot delegate this to AI. Opaque AI systems – those providing recommendations without disclosing reasoning or enabling rapid verification – generate “chronic doubt:” the recurring question of whether the pathologist missed something the AI detected (1,20). Chronic doubt is cognitively expensive, manifesting as repetitive case review, second-guessing of initial assessments, and anxiety about potential errors. Over time, it depletes brain capital and contributes to professional burnout. Preservation of signing authority requires spatially localized, verifiable outputs: a pathologist can rapidly confirm “suspicious focus at coordinates (2450, 3800) in section A3” but cannot efficiently verify “71% probability of cancer.”

One approach to preserving signing authority is illustrated by Paige Prostate (13,21). This FDA-authorized tool provides localized (X, Y) coordinates of regions suspicious for adenocarcinoma, enabling direct navigation within the existing diagnostic workflow. In clinical validation across 16 pathologists reviewing 610 biopsies, Paige Prostate reduced cancer detection errors by 70% and increased sensitivity by 8 percentage points across all histologic grades (13). Real-world deployment data further showed approximately 20% reduction in slide reporting time, 20% fewer ancillary immunohistochemistry studies ordered, and a 40% reduction in second-opinion consultation requests (22) – benefits attributable in part to the psychological confidence conferred by spatially verifiable AI output.

### Criterion 3: Evidence Gap Analysis

Clinical decision-making frequently involves diagnostic uncertainty: equivocal morphology, borderline quantitative assessments, or cases where multiple diagnoses remain plausible. These unresolved questions create persistent cognitive burden through the Zeigarnik effect, whereby incomplete tasks occupy working memory more prominently than resolved ones (7,23). Evidence gaps consume brain capital both during case review (through repeated mental revisitation of uncertain findings) and through follow-up work (ordering ancillary studies, collegial consultation, literature review). AI tools that close evidence gaps – providing precise quantitative measurements, standardizing assessments, or highlighting discriminating features – directly reduce this burden. Tools that create new uncertainty (“why does this AI disagree with my assessment?”) are counterproductive.

One illustrative example of evidence gap closure in clinical use is Lunit SCOPE PD-L1 (14). PD-L1 expression assessment is critical for immunotherapy eligibility across multiple cancer types but shows significant inter-observer variability near clinical decision thresholds (1%, 5%, and 50%). In validation studies, Lunit reduced reading time by 32% while maintaining concordance with reference standards. Rather than requiring pathologists to maintain a running mental tally across multiple high-power fields, the AI provides a precise, reproducible measurement – definitively closing the evidence gap and allowing the pathologist to proceed without lingering uncertainty. This principle extends to Ki-67 quantification (24), mitotic counts, and tumor cellularity estimates, each representing a domain where AI-provided precision closes gaps that would otherwise require prolonged deliberation.

### Criterion 4: Verification and Stress-Testing

All AI systems make errors; the clinically critical question is whether errors can be detected efficiently and reliably. If verification requires equivalent effort to performing the original task without AI, the tool provides no net cognitive benefit – it merely redistributes work. Verification feasibility depends on error rate, error severity, detectability, and verification time; a tool with higher error rates but obviously detectable failures may be more clinically useful than one with lower error rates but subtle errors. Effective tools enable spot-checking rather than an exhaustive review, flag cases where the AI has low confidence, and fail gracefully with obvious rather than subtle errors. Vendors should publish stress-test results across artifact categories – tissue artifacts, staining variations, scanning defects, rare morphological patterns – not merely average performance on curated test sets.

One published approach to addressing the verification challenge for virtual staining is the AQuA (Assessing Quality in AI-generated images) framework (15,25,26). Virtual staining uses AI to predict special stains from H&E images but risks generating plausible-appearing but incorrect staining patterns. AQuA employs a secondary AI model to detect such “hallucinations” by comparing image features to known artifact patterns, achieving 99.8% detection accuracy at 1-2 seconds per field. This matches the timescale of clinical workflows: a pathologist can assess AQuA output during normal slide review. Without such a companion tool, verifying virtual stains would require obtaining physical stains for every case, negating the technology’s benefits entirely. AQuA illustrates a broader design principle: high-stakes primary AI systems may require purpose-built verification companions as a prerequisite for safe clinical deployment.

### Criterion 5: Resource and Opportunity Cost

Every AI implementation requires investment in software licensing, computing infrastructure, training, and workflow integration. These investments are justified only by a positive return: a net increase in available brain capital or improved diagnostic quality. Return should be assessed at the system level rather than per case: a tool saving 2 minutes per case but requiring 15 minutes of daily troubleshooting provides minimal net benefit. Meaningful metrics include time per case, rates of ancillary study ordering, turnaround times, pathologist satisfaction, and burnout indicators.

One illustrative example of quantifiable positive return through prostate biopsy AI implementation is provided by ZAS Hospitals in Antwerp (10). AI pre-screening and case prioritization decreased turnaround times by an average of 9 hours. Ancillary immunohistochemistry use decreased by approximately one-third, yielding both cost savings and reduced interpretive burden. Pathologists reported improved workflow satisfaction, with cognitive resources redirected toward genuinely challenging cases rather than high-volume routine searches. Financial break-even was achieved within 18 months when accounting for reagent savings, improved throughput, and deferred pathologist hiring. Conversely, AI implementations commonly fail to achieve positive return through hidden costs: poor interoperability requiring manual data transfer, high false-positive rates demanding extensive alert dismissal, or specialized hardware with poor utilization – each increasing rather than decreasing extraneous cognitive load.

## Discussion

The five calibration criteria – Contextual Literacy, Responsibility, Evidence Gap Analysis, Verification, and Opportunity Cost – are not independent criteria but interconnected dimensions of AI tool evaluation. A tool may excel on one dimension while failing on another, and overall clinical utility depends on the aggregate profile. A hypothetical lymphoma classification tool with strong contextual literacy and effective evidence gap closure that fails on responsibility and verification may produce a net negative outcome: diagnostic suggestions the pathologist cannot efficiently verify generate chronic doubt that consumes more brain capital than the tool saves. Conversely, a tool with more modest technical performance that excels across all five criteria may prove more valuable in practice – a finding that challenges the conventional AI development paradigm prioritizing technical metrics above all else.

This framework aligns with emerging regulatory requirements for medical AI. The European Union AI Act mandates transparency, human oversight, and explainability for high-risk AI systems – elements directly addressed by Criteria 2 and 4 (27,28). US Food and Drug Administration guidance on clinical decision support similarly emphasizes that AI tools should not obscure human judgment or create over-reliance, concerns at the core of our framework’s emphasis on signing authority and verification feasibility. Pathology departments adopting this framework position themselves ahead of regulatory requirements, demonstrating structured due diligence beyond technical validation.

Several limitations warrant acknowledgment. The framework is primarily qualitative and would benefit from quantitative scoring instruments enabling comparisons across tools and tracking improvement over time. It addresses individual AI tools rather than the cumulative cognitive load of multiple simultaneous AI systems within a single workflow – an increasingly relevant concern as AI adoption grows. The framework is grounded in CLT and implementation science validated through case study analysis, but lacks prospective randomized controlled trials comparing framework-guided with conventional implementation; such trials would strengthen its evidence base. Finally, the framework assumes pathologist-primary diagnostic authority; it does not directly address emerging debates about AI-primary models with pathologist oversight.

Pathologists are well-positioned to serve as active evaluators and gatekeepers of AI technology rather than passive recipients. Cognitive sustainability represents an immediate practical priority in an environment of workforce shortages and expanding diagnostic responsibilities. By evaluating AI tools through the lens of cognitive impact – asking how tools integrate context, whether outputs are rapidly verifiable, how the model handles artifacts, and what the cognitive return on investment is – pathologists exercise a form of expertise that no amount of engineering can replicate. Importantly, this evaluative role does not require pathologists to become programmers or machine learning practitioners. The five criteria in this framework are clinical criteria: they ask whether an AI tool respects diagnostic context, supports medical-legal accountability, closes genuine evidence gaps, fails gracefully, and returns cognitive value. These are questions that clinicians are uniquely positioned to answer, and doing so well is a high-value contribution to AI development. Pathologists who articulate precise failure modes, define the clinically acceptable boundaries of automation, and specify the contextual information an AI must integrate are doing the foundational work that separates tools that succeed in the research laboratory from tools that succeed in the diagnostic clinic (4,29).
